# 2130. Safety and Pharmacokinetics of SPR206 in Subjects with Varying Degrees of Renal Impairment

**DOI:** 10.1093/ofid/ofad500.1753

**Published:** 2023-11-27

**Authors:** Jon Bruss, Justin C Bader, Kamal Hamed

**Affiliations:** Spero Therapeutics, Cambridge, Massachusetts; Spero Therapeutics, Inc., Cambridge, Massachusetts; Spero Therapeutics, Inc., Cambridge, Massachusetts

## Abstract

**Background:**

SPR206 is a novel polymyxin derivative with potent *in vitro* activity against susceptible and multidrug-resistant strains of *Acinetobacter baumannii*, *Pseudomonas aeruginosa*, *Klebsiella pneumoniae*, *Escherichia coli*, and *Enterobacter* species. In nonclinical studies, SPR206 had similar or greater activity than polymyxin B with an improved safety profile.

**Methods:**

We evaluated the safety, tolerability, and pharmacokinetics (PK) of SPR206 in healthy subjects with normal renal function and subjects with various degrees of renal impairment (RI) or end-stage renal disease (ESRD) on hemodialysis (HD). All subjects except those with ESRD on HD received a single 100 mg dose of SPR206 as a 1-hour intravenous (IV) infusion. Subjects with ESRD on HD received a 100 mg IV dose within 2 hours after HD on Day 1 (Period 1) and 1 hour before HD on Day 5 (Period 2).

**Results:**

Among 37 subjects, no deaths, treatment-related serious adverse events (AEs), or discontinuations due to AEs were reported. Paresthesias were the most common treatment-related AEs. Systemic exposure to SPR206 increased as RI worsened, with mean AUC

_0-last_ of 39% to 239% greater with mild to severe RI vs. healthy subjects (Figure). Renal clearance (CL) decreased (1.2 L/h to 0.13 L/h) with decreasing renal function, and plasma CL was 29% to 76% lower with RI vs. healthy subjects. In subjects with ESRD on HD, mean AUC_0-last_ was 372% greater and mean CL was 87% lower in Period 1 (nondialyzed) vs. healthy subjects. In Period 2 (dialyzed), AUC_0-last_ was 131% greater and CL was 75% lower vs. healthy subjects. In subjects with ESRD on HD, SPR206 systemic exposure (AUC_0-last_) decreased by 51% and CL increased by 92% during dialyzed vs. nondialyzed conditions. AUC was negatively correlated and CL positively correlated with estimated glomerular filtration rate (eGFR) (p< 0.0001). SPR206 was mostly excreted in urine within 12 hours in healthy subjects and subjects with mild RI, but excretion was slower in subjects with moderate and severe RI. HD was effective in removing SPR206 from the circulation.

**Figure:**
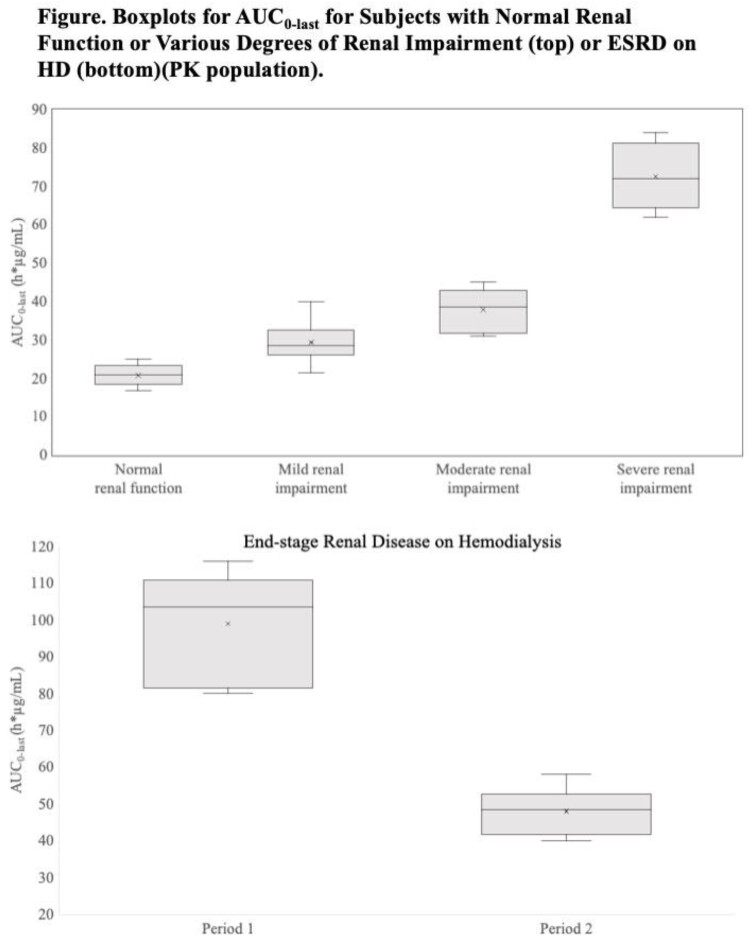

**Conclusion:**

In summary, SPR206 appeared to be safe and well tolerated in subjects with varying degrees of RI including those with ESRD on HD. These results will inform dose adjustments in future studies for patients with various degrees of RI including those with ESRD on HD.

**Disclosures:**

**Jon Bruss, M.D.**, Spero Therapeutics, Inc.: employee **Justin C. Bader, PharmD**, Spero Therapeutics, Inc.: former employee **Kamal Hamed, MD, MPH**, Spero Therapeutics, Inc.: current employee

